# Assessing decision-making capacity in clinical practice in Norway: a qualitative exploration of stakeholder perspectives

**DOI:** 10.1186/s12888-025-07161-z

**Published:** 2025-10-10

**Authors:** Jacob Jorem, Reidun Førde, Tonje Lossius Husum, Jørgen Dahlberg, Reidar Pedersen

**Affiliations:** 1 Institute of Health and Society, University of Oslo, Oslo, Norway; 2 Department of Health Care Policy, Harvard Medical School, Boston, USA; 3 Department of Health Policy and Management, Columbia University Mailman School of Public Health, New York, USA; 4 Faculty of Health Sciences, Oslo Metropolitan University, Oslo, Norway

**Keywords:** Mental health policy, Psychiatry and law, Decision-making capacity, Capacity-based health law, Involuntary care, Mental health services, Clinical assessment, Ethics

## Abstract

**Background:**

Decision-making capacity (DMC) is a widely used criterion in health law, but assessments pose challenges in practice. In Norway, lacking DMC became an additional criterion for involuntary care and treatment following comprehensive amendments to the Mental Health Care Act in 2017. Contrary to the amendments’ objectives, involuntary care rates have continued to increase after an initial reduction in 2017. Assessing DMC typically involves four abilities: understanding, reasoning, appreciating relevant information, and communicating a choice. This four abilities model was introduced to aid in DMC assessments. With limited assessment experience pre-2017, the Norwegian context offers valuable insights into how stakeholders integrate DMC into clinical practice over time. This study aimed to explore how DMC was assessed in clinical practice following the introduction of a capacity-based mental health law governing involuntary care and treatment in Norway.

**Methods:**

In 2018, semi-structured interviews and focus groups were conducted with 44 key stakeholders, including psychiatrists, specialists in clinical psychology, general practitioners, and lawyers in supervisory bodies (the Control Commission and County Governor). In 2022–23, 21 of these participants took part in individual follow-up interviews. The interviews were transcribed and thematically analysed.

**Results:**

Data analysis generated three themes with subthemes: (1) DMC assessments primarily relied on the four abilities model in specialist care, experiencing gradual clinical adaptation with decreased importance, and exhibited variations in quality, particularly in primary care; (2) several challenges in DMC assessments, including lack of training in applying the four abilities model, ownership, continuity of care, information, and patient cooperation, with certain patient groups posing particular challenges, such as those with manic symptoms, substance misuse, and severe eating disorders; and (3) quality assurance measures needed, including systematic training and tools to improve assessment quality.

**Conclusions:**

Assessing DMC involves variations and several challenges across the healthcare system. While the four abilities model served as a primary basis of DMC assessments in specialist care, systematic training, validated tools, and further research seem needed to improve assessment quality and better understand factors influencing assessments. Recognising the complex interplay between legal, health service, and societal factors when implementing health law reforms seems crucial for achieving their objectives.

**Supplementary Information:**

The online version contains supplementary material available at 10.1186/s12888-025-07161-z.

## Background

Decision-making capacity (DMC) is a widely used criterion in health law, but assessments pose challenges in practice [1]. While DMC has long played a key role in assessing informed consent in healthcare, the lack of DMC has become an increasingly important criterion for involuntary care and treatment in several jurisdictions [2], aligning with human rights developments [3]. Lacking DMC implies an inability to give valid consent [4] and is central to the ongoing international debate over appropriate criteria for involuntary care and treatment [3]. DMC assessments seek to balance respecting patient autonomy with protecting patients from the potential consequences of treatment decisions, posing complex clinical, ethical, and legal challenges [5].

A commonly used functional model for assessing DMC includes four abilities: understanding, reasoning, appreciating relevant information, and communicating a choice [5–7]. This four abilities model provides the framework for several DMC assessment tools, such as the MacArthur Competence Assessment Tool for Treatment (MacCAT-T) [7]. Systematic reviews indicate that DMC assessments can be conducted reliably and that most mental healthcare inpatients can make treatment decisions [8, 9]. However, studies suggest that DMC assessments remain challenging, including inherent issues with applying the four abilities model in practice [1, 10–12], determining whether decisions are driven by illness [1], and assessing patients with affective disorders [11].

**Fig. 1:**
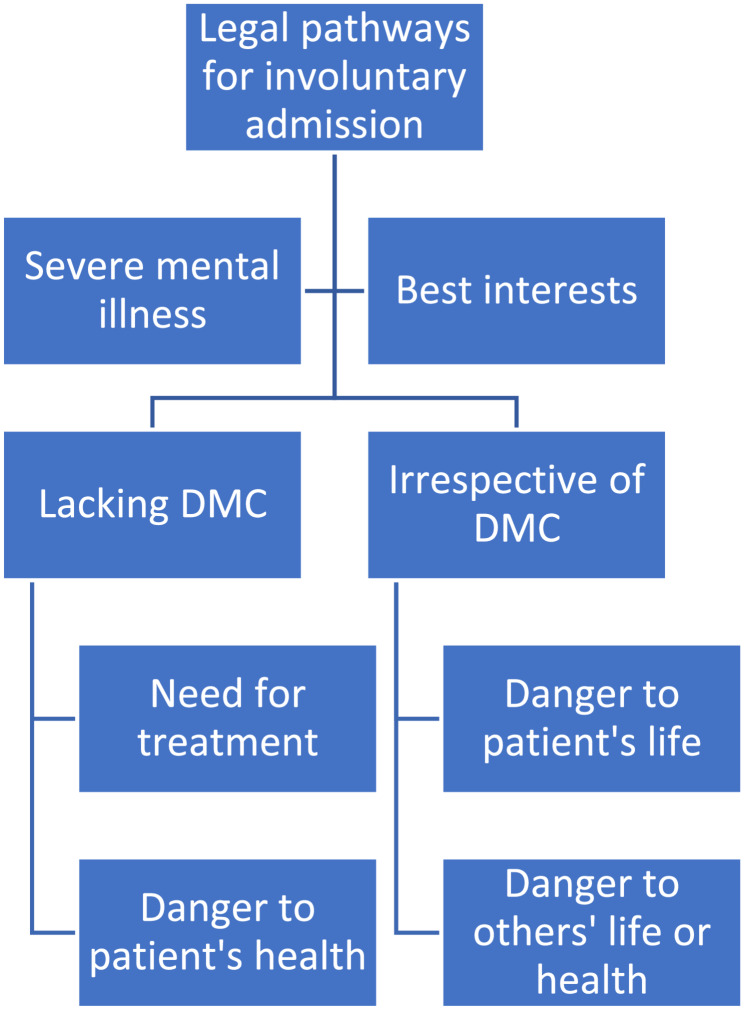
Key criteria within the two main legal pathways for involuntary admissions, either lacking or irrespective of DMC, specified in MHCA, Section The need for quality assurance measures in DMC assessments

In 2017, a lack of DMC was introduced as an additional criterion for involuntary care and treatment in the Norwegian Mental Health Care Act of 1999 (MHCA) [13]. However, the DMC criterion does not apply in cases of danger to the patient’s life or others’ life or health. This implies that competent patients who pose a danger to their health cannot be involuntarily admitted or treated. For involuntary care and treatment decisions under the MHCA, several other criteria must be met, including severe mental illness, a need for treatment or danger to the life or health of oneself or others, and the patient’s best interests (see Fig. 1) [14].

Combined with the comprehensive 2017 amendments improving patients’ procedural rights, lacking DMC was introduced to increase patient autonomy and reduce involuntary care and treatment use in Norway [3]. By effectively narrowing the need for treatment criterion, introducing DMC involved stricter standards for involuntary care and treatment decisions. Notably, the amendments intended to shorten the duration of Community Treatment Orders (CTOs) [15]. Although the amendments appear to have raised awareness of patient autonomy among health professionals [16–18], Norway’s involuntary care and treatment rates have continued to increase, following pre-amendments trends, after an initial reduction in 2017 [15, 19]. Over the past decade, growing pressures on the mental healthcare system – exacerbated by reduced inpatient bed availability and increased demands on primary health services – may have contributed to limited access to voluntary care options and increased morbidity and involuntary care rates [14, 15, 20–22].

Under Norwegian health law, physicians in primary care and outpatient clinics evaluate whether the criteria under the MHCA, including lacking DMC, are met when referring patients to involuntary hospitalisations in specialist healthcare (see Fig. 1). Within 24 h of the patient’s admission, psychiatrists or specialists in clinical psychology at the hospital make an initial decision regarding involuntary care in accordance with the MHCA. Involuntary care follow-up options include either inpatient clinics or outpatient clinics under CTOs. Psychiatrists also conduct DMC assessments as part of involuntary treatment decisions, which require observing the patient for five days following an involuntary care decision. Additionally, supervisory bodies (the Control Commission and County Governor) conduct DMC assessments as part of their legal review of criteria under the MHCA following appeals by patients, their families, or public authorities. To support DMC assessments, the four abilities model was introduced in the circular to the MHCA in 2017 [15].

Since 2017, research indicates that Norwegian clinicians have expressed concern about the quality of DMC assessments [17], noting limited assessment experience before the amendments [23]. In addition to the challenges described internationally, specific Norwegian ones include the strict evidentiary requirement that patients “obviously” lack DMC, leaving little room for uncertainty in assessments [14, 15]. Another study on Norwegian experiences highlights that DMC assessments may be challenged by time constraints, fluctuating patient conditions, substance misuse, and limited patient information [17]. Moreover, some studies recommend systematic assessment training and tailored education to improve clinical and legal expertise [12, 24]. However, Aid to Capacity Evaluation (ACE) is the only validated assessment tool available in Norwegian and is primarily designed for somatic clinical settings [15].

Despite DMC’s growing international significance, empirical research on assessments in clinical practice remains scarce. With limited assessment experience pre-2017, the Norwegian context offers valuable insights into how stakeholders integrate DMC assessments into clinical practice over time. Since lacking DMC is a criterion for involuntary care and treatment, examining assessment quality is crucial for safeguarding patient rights and enabling effective healthcare provision. This study aimed to explore how DMC is assessed in clinical practice following the introduction of a capacity-based mental health law governing involuntary care and treatment in Norway.

## Methods

This study used qualitative semi-structured interviews and focus groups with key stakeholders, including psychiatrists, specialists in clinical psychology, general practitioners, and lawyers in supervisory bodies (the Control Commission and County Governor). It was part of a larger qualitative research project at the Institute of Health and Society at the University of Oslo, and is one of three studies exploring various aspects of introducing a capacity-based mental health law in Norway in 2017, including its impact and factors influencing DMC assessments [14, 23]. Patient and family participants were also interviewed for the larger research project. However, questions about DMC assessments in the interview guide were only directed to clinical and supervisory body participants. Thus, the participants in this study are limited to the latter two groups.

This study addressed two research questions: First, how is DMC assessed following the introduction of a capacity-based mental health law governing involuntary admission and treatment, and what are the assessment challenges? Second, is there a need for training and tools to assess DMC?

### Study design

Using a qualitative study design, semi-structured interviews and focus groups were conducted in 2018 with 44 stakeholders. In 2022–23, 21 of these participated in individual follow-up interviews.

### Research group

The five researchers (JJ, RF, TLH, JD, and RP) had backgrounds in psychology, psychiatry, medicine, law, and ethics. All researchers had experience with DMC, involuntary mental healthcare, and teaching in different settings. While the interviewers avoided sharing characteristics before and during interviews, participants may have been familiar with them. However, the research group made efforts to prevent interviewers from having established relations with participants before the interviews. The research group evaluated researchers’ influence on study validity through ongoing reflexive practices, including researcher positionality [25].

### Setting

The individual interviews and focus groups occured in both urban and rural locations across Norway, primarily in participants’ offices or other locations of their choosing. One of the interviews in 2022–23 was conducted virtually.

### Sampling and recruitment

Clinicians and lawyers experienced with conducting DMC assessments in mental healthcare were purposively sampled in 2018 [26]. Participants with experience before and after 2017 in involuntary care and treatment decisions were seen as particularly relevant. Clinical participants were selected from various parts of the healthcare system, including general practice, urgent care clinics, outpatient clinics, and psychiatric wards.

Participants were sampled from four geographic areas in Norway, reflecting variations in involuntary care rates based on national health registry data [27, 28]. Three urban and one rural area was selected. Clinical participants were recruited from all four areas. Due to challenges in recruiting relevant participants and some declining to participate, supervisory body participants were only recruited from two areas. 

In 2018, participants were recruited via national organisations representing key stakeholders using a reputational sampling method. These organisations recommended participants experienced in DMC assessments from the four selected geographic areas. Each new participant would recommend other relevant participants. 

 Participants were first contacted via email and subsequently by phone calls in both interview rounds. Throughout the recruitment process, the research group aimed to ensure sufficient variation among participants in each group and reach data saturation [29].

As part of the larger research project at the University of Oslo, a seven-member stakeholder advisory group was recruited based on recommendations from national organisations representing all stakeholder groups. This advisory group gave valuable feedback for each study in the project. While all supervisory body and some clinical participants were recruited based on recommendations from the advisory group, additional clinical participants were recruited based on recommendations from other participants.

While aiming to reinterview as many 2018 clinical and supervisory body participants as possible in 2022–23, inclusion required a minimum of two years of relevant experience since the 2018 interviews. While 21 of the same participants took part in 2022–23, the remaining 23 did not to participate due to various reasons: failing to meet the inclusion criterion (6 participants), time constraints (7 participants), health issues (2 participants), retirement (3 participants), or communication challenges (5 participants).

### Individual interviews and focus groups

44 participants were interviewed individually (36 participants) or in focus groups (8 participants)in 2018. Three separate focus groups were conducted, including specialist care (3 participants), primary care (2 participants), and supervisory body (3 participants) participants. This approach was taken at the participants' request and aimed to improve their comfort in speaking freely. The duration of individual interviews was approximately 60 min, while the focus groups extended to 90 min. The same semi-structured interview guide – developed before the first data collection round – was used consistently for individual and group settings. Either TLH or JD led the three focus groups. The 36 individual interviews were conducted by one of four researchers (JJ, TLH, JD, RP), with JJ and RP conducted two individual interviews jointly.

In 2022–23, individual follow-up interviews were conducted with 21 of the same participants as in 2018. The 2022–23 interview instructions asked participants to reflect on changes since the last interview or focus group. JJ conducted 18 interviews, JJ and RP conducted two interviews jointly, and RP conducted one independently. Specialist and primary care participants in 2022–23 had at least five years of clinical experience, with 83 per cent having 15 or more years. Several interviewed clinicians held managerial roles, managed emergency medical responsibilities, and worked across various departments, including urgent care centres, district psychiatric centres, geriatric psychiatry wards, and secure units. All supervisory body participants in 2022–23 had a minimum of five years of legal experience with the MHCA, either in the local Control Commission or County Governor (see Table 1).

**Table 1:** Role and number of participants in the 2018 individual interviews and focus groups as well as the 2022–23 individual follow-up interviews

	2018 interviews and focus groups			2022–23 interviews
Role	Population (*N* = 44) (%)	Individual interviews (*N* = 36) (%)	Focus groups (*N* = 8) (%)	Population (*N* = 21) (%)
Specialist care^a^	*27 (61)*	*24 (55)*	*3 (7)*	*16 (76)*
Primary care^b^	*12 (27)*	*10 (23)*	*2 (5)*	*2 (10)*
Supervisory bodies^c^	*5 (11)*	*2 (5)*	*3 (7)*	*3 (14)*

^a^Specialist care participants included specialists in psychiatry and clinical psychology

^b^Primary care participants included general practitioners, physicians at urgent care centres, and district medical officers

^c^Supervisory body participants included members from the Control Commission (Kontrollkommisjonen) and officials from the County Governor' offices (Statsforvalteren)

The individual interviews and focus groups were audio-recorded and transcribed. The research group guided the research assistants in verifying the correspondence between the audio recordings and transcripts. Field notes the research group took during and after the individual interviews and focus groups provided context for the data analysis.

This study analyses the responses of participants to the following questions in the interview guide (Supplementary Material 1):

1. How is DMC assessed? How do you evaluate the quality of the assessments? Are there any challenges related to these assessments? Do you find that DMC is assessed similarly?
2. What is the need for training and tools to assess DMC?

### Data analysis

Four researchers (TLH, RP, JD, and JJ) initially analysed the 2018 individuals interviews and focus groups. The data from both interview rounds were reanalysed independently by JJ, RF, and RP after the 2022–23 interviews were conducted. After familiarising themselves with the audio recordings and transcripts from 2018 and 2022–23, JJ, RF, and RP sorted the data into initial codes. These initial codes were adjusted by comparing the codes of each researcher. Analysis of the coded data generated potential themes and subthemes. The data was reanalysed and the themes and subthemes were further refined in an iterative process [25, 30–32]. All members of the research group contributed to the data analysis. NVivo (Windows Release 1.6.1) was used for data management. Changes in participant experiences over time were identified through comparative analysis of the two interview rounds. Preliminary findings of this study were discussed with the stakeholder advisory group.

### Ethics

Written information about the research project was provided to participants before the interviews, which was reiterated verbally at the start of the interviews. Written informed consent was provided by participants. Access to the data was restricted to study personnel, and it is securely stored on the University of Oslo’s services for sensitive data. The authors assert that all procedures contributing to this work comply with the ethical standards of the relevant national and institutional committees on human experimentation and with the Helsinki Declaration of 1975, as revised in 2008. All procedures involving human subjects were approved by the Regional Committee for Medical and Health Research Ethics (REK South-East (2018/488/REK South-East)), the Data Protection Officer at the University of Oslo, and the Norwegian Agency for Shared Services in Education and Research (Sikt (400975)).

## Results

The analysis of the 2018 and 2022–23 interview data generated three themes with six subthemes and corresponding codes (see Table 2). While the different groups of participants generally shared similar views, differences among them are highlighted in the presentation of results. Illustrative quotes are from the 2022–23 interviews when the participants had more experience with DMC assessments and the broader legislative amendments. Thus, the 2018 interviews became a key reference point for exploring changes in DMC assessments over time.

**Table 2:** Themes, subthemes, codes, and illustrative quotes from the 2022–23 interviews regarding assessing DMC following the introduction of a capacity-based mental health law in Norway in 2017 (see Table 3 for more illustrative quotes)

Theme	Subtheme	Code	Quote (type of participant, participant number, quotation number)
*1. Evolving practices and variations in DMC assessments*	1 A. The four abilities model as a basis for DMC assessments	Mainly in specialist care	*“I believe I am the only one in my office of several general practitioners*,* who is familiar with the four abilities model.” (Primary care participant*,* 26*,* Q1)*
	1B. Changes in DMC assessments over time	Gradual clinical adaptation	*“In the beginning*,* DMC assessments were closely tied to the four abilities model. It is a common developmental trajectory that initially*,* when lacking knowledge*,* one adheres closely to what one has been taught. Over time*,* it becomes possible to develop in line with practical experience. […] Eventually*,* one can place more emphasis on having a good conversation and less on asking the right questions while still eliciting the necessary information for a thorough assessment.” (Specialist in clinical psychology*,* 9*,* Q2)*
		Decreased importance	*“When DMC was introduced*,* one would think it was the sole criterion for involuntary care as it received considerable emphasis. Now I perceive it as one of several criteria.” (Supervisory body participant*,* 6*,* Q3)*
	1 C. Variations in DMC assessments	Variations in quality	*“DMC is far from being uniformly assessed. These assessments are undoubtedly influenced by a multitude of factors.” (Psychiatrist*,* 3*,* Q4)*
*2. Challenges to DMC assessments*	2 A. Barriers to implementation and quality assessments	Lack of training in applying the four abilities model	*“There has been a lack of training*,* particularly in primary healthcare services. They were quite unprepared for the legislative amendments. So*,* understanding how to assess DMC has taken a considerable amount of time. We are probably not yet at a point where we fully comprehend it.” (Psychiatrist*,* 3*,* Q5)*
		Lack of ownership	*“Many of us do not feel a sense of ownership regarding the requirement to assess DMC*,* and we do not consider it as particularly meaningful or significant. […] We believe that it is almost more of a nuisance than a necessity in our clinical work.” (Psychiatrist*,* 1*,* Q6)*
		Lack of continuity of care, information, and patient cooperation	*“If the patient refuses to communicate with me*,* which happens quite often*,* I have very little information. This makes it challenging to make the assessment. I need information to conduct the assessment.” (Psychiatrist*,* 19*,* Q7)*
	2B. Patient groups where assessing DMC was particularly challenging	Manic symptoms	*“I have found DMC assessments of patients with manic symptoms to be quite complex. […] These patients were challenging even before the legislative amendments*,* but perhaps it has become more pronounced since then.” (Psychiatrist*,* 7*,* Q8)*
		Substance misuse	*“Substance misuse can complicate assessments of DMC*,* as it can both contribute to the loss of DMC in one or more areas and trigger other problems that limit DMC.” (Psychiatrist*,* 21*,* Q9)*
		Severe eating disorder	*“Assessing DMC of patients with […] eating disorders can be challenging. Patients with manic symptoms and eating disorders are often assessed competent if one takes what they say at face value. Therefore*,* it is crucial to be much more attentive to avoid overestimating the patient’s ability to make a choice. They typically downplay their own deterioration and symptoms*,* possibly not recognizing the extent of their challenges.” (Specialist in clinical psychology*,* 9*,* Q10)*
*3. The need for quality assurance measures in DMC assessments*	3. Training and tools in DMC assessments	Need for training	*“I have not received any training or guidance in DMC assessment. I have had to take responsibility for that myself.” (Primary care participant*,* 26*,* Q11)*
		Need for tools	*“I believe there is a significant need for training on simple tools for assessing DMC*,* such as the four abilities model.” (Primary care participant*,* 23*,* Q12)*

### Evolving practices and variations in DMC assessments

#### The four abilities model as a basis for DMC assessments

*“I use the four abilities model extensively. I always revert to these four elements when dealing with very complex cases. For simpler cases*,* it’s more straightforward*,* like “No insight”.” (Psychiatrist*,* 1*,* 2022–23*,* Q13)*

Specialist care and supervisory body participants noted widespread adoption of the four abilities model for DMC assessments in specialist care after 2017. In the 2022–23 interviews, they noted a gradual integration of the four abilities model into clinical practice. This model was also used explicitly to justify a lack of DMC in involuntary care decisions in medical records, as noted by specialist care and supervisory body participants. Some raised concerns about the four abilities model compromising the thoroughness of DMC assessments. Although they used the four abilities model, it was integrated into clinical evaluations despite lacking a clear assessment structure. This included clinicians not disclosing their understanding before applying this model and not informing the patient when DMC was being assessed. Primary care participants described limited familiarity with the four abilities model both in acute care and general practice.

#### Changes in DMC assessments over time

*“I believe I have shifted towards a bit more pragmatism*,* both in written documentation and perhaps in the assessments themselves. […] There was a return to allowing more clinical discretion and perhaps more overarching formulations.” (Psychiatrist*,* 10*,* 2022–23*,* Q14)*

Several specialist care participants noted developments in DMC assessments from 2018 to 2022–23, observing a gradual clinical adaptation of the assessments. Despite initial challenges, several specialist care participants highlighted improved DMC assessment documentation in the 2022–23 interviews. Moreover, they noted a shift towards more clinically relevant information and language instead of generic legal phrasing. These developments correspond with a shift in the perspectives of several participants on the importance of DMC, from being a focal point in the 2018 interviews to becoming merely one of several criteria assessed for involuntary care in 2022–23. Some believed that reduced emphasis on DMC resulted in assessments becoming less comprehensive and more conclusive with less justification since 2017. Several noted a gradual decrease in the time spent clinically assessing DMC. In parallel, they highlighted an increase in the time dedicated to documenting involuntary care decisions in patient records since 2017.

#### Variations in quality of DMC assessments

*“I believe that the quality of DMC assessments varies significantly*,* and I think they are done differently. This is because a good*,* standardised way of doing these assessments has not been found.” (Psychiatrist*,* 19*,* 2022–23*,* Q15)*

Several specialist care and supervisory body participants highlighted variations in DMC assessments across healthcare sectors, with less variation in specialist care. Some noted differences in assessment quality by ward type, with long-term wards often conducting more thorough assessments than acute wards due to having more time and access to patient information. However, experience from managing involuntary care in acute psychiatric wards could reduce variations, according to some. They emphasised discussions with colleagues as vital for calibrating assessments. While supervisory body participants observed improved DMC assessments in specialist care since 2017, they found no corresponding development in primary care. For example, the evidentiary requirement of “obviously” lacking DMC was emphasised in specialist care but overlooked in primary care, potentially resulting in variations in assessments and referrals to involuntary admissions.

### Challenges to DMC assessments

#### Barriers to implementation and quality assessments

*“If there is lack of time to talk or assess the patient*,* if it is very busy*,* and you have to make a quick assessment*,* it often results in a poor DMC assessment.” (Psychiatrist*,* 2022–23*,* 17*,* Q16)*

Several specialist care participants highlighted challenges with applying the four abilities model in clinical practice. They found it difficult to differentiate between the four abilities, particularly reasoning and appreciating. Several noted the initial emphasis by the health authorities on differentiating DMC from the patient’s insight into their illness. However, insight seemed to gradually have regained attention, with participants highlighting similarities with DMC in the 2022–23 interviews. Supervisory body participants noted that deficiencies in the ability to reason about mental illness often led to assessments of lacking DMC. Some specialist care participants questioned using the ability to appreciate relevant information when patients disagreed with clinicians’ understanding, finding it problematic to base DMC assessments solely on a medical understanding. These experiences reveal misconceptions about DMC assessments, which will be discussed further in the Section Challenging to assess DMC and the need for quality assurance.

Specialist and primary care participants highlighted time constraints for communication and observation as a challenge in conducting valid DMC assessments. They stressed that assessment validity often relied on establishing trust with the patient to gain insight into their inner world. Some noted the risk of overestimating DMC without observing patients over time. Supervisory body participants highlighted additional challenges when patients learned to temporarily “appear competent,” which could skew assessments. Several participants found that inadequate patient information or limited familiarity with patients posed challenges, particularly during acute phases. Furthermore, lack of patient cooperation posed challenges, according to some, often requiring assistance from trusted individuals like family or other health professionals. Many also mentioned increased bureaucratisation of clinical practice, mainly related to other aspects of the 2017 amendments, which limited the time available for assessing DMC. Several participants emphasised that these barriers to implementation and quality assessments increased the risk of overestimating DMC. However, they underscored that many of these challenges also applied to assessing other criteria for involuntary care.

#### Patient groups where assessing dmc was particularly challenging

*“I think that assessing DMC of patients with bipolar disorder or manic symptoms is more challenging. There is a potential for greater harm if involuntary care is lifted prematurely.” (Supervisory body participant*,* 2022–23*,* 6*,* Q17)*

Several participants emphasised significant challenges in assessing DMC in patients with manic symptoms due to their fluctuating conditions and tendency to conceal behaviour. They highlighted that fluctuating illness severity made it difficult to determine which status to base their assessments on. Moreover, they noted that these patients often minimised their display of symptoms, potentially leading to an overestimation of DMC. High cognitive functioning among some of these patients further complicated assessments based on the four abilities model, requiring more time for observation and assessment, as well as the collection of comprehensive information on daily functioning from additional sources. To prevent severe consequences if left untreated, they described a gradual lowering of the threshold for lacking DMC for patients with manic symptoms since 2017 in the 2022–23 interviews.

Several specialist care and supervisory body participants noted similar challenges in assessing DMC in patients with comorbid substance misuse and often fluctuating conditions. These participants faced challenges in assessing DMC over time due to such fluctuations. Moreover, they emphasised that the potential consequences of discharge, particularly relapse in substance misuse, often overshadowed other considerations in assessments involving this patient group.

Several specialist care participants raised concerns about competent patients with severe eating disorders not adhering to treatment and posing risks to themselves, not amounting to the exception of danger to the patient’s life. Assessing DMC for these patients often proved challenging due to ambiguity about whether treatment decisions were the patient’s own or driven by their illness. Moreover, they expressed concerns about deteriorations among competent patients with severe eating disorders going unnoticed. However, several participants stressed that assessing the criteria for involuntary care in patients with manic symptoms, comorbid substance misuse, and severe eating disorders had been challenging even before the 2017 amendments.

### The need for quality assurance measures in DMC assessments

*“There is considerable variation*,* and I believe that there is a need to elevate the overall knowledge level to approach assessments more uniformly.” (Specialist in clinical psychology*,* 25*,* 2022–23*,* Q18)*

According to specialist care participants, validated tools for DMC assessments were rarely used. In challenging cases, a few participants reported using ACE. In 2018, several participants emphasised the need for validated tools to assess DMC and more comprehensive assessment training, pointing to the shortcomings of the four abilities model in complex assessments. In 2022–23, some reiterated this need for validated tools to ensure assessment quality. However, the majority regarded the four abilities model as adequate despite its challenges, emphasising their preference to avoid a validated tool that could disrupt clinical communication. This development may be related to the gradual clinical adaptation of DMC assessments (see the Section on Changes in DMC assessments over time). However, none of the participants recognised that the four abilities model is not an assessment tool, nor acknowledged its lack of validation in a Norwegian context and the potential implications for assessment quality.

Specialist care participants noted varied training initiatives initially aimed at educating health professionals about the legislative amendments, including DMC assessments. However, they observed a gradual decline in training initiatives by 2022–23, mainly relying on individual efforts. They pointed to high turnover rates of employees, particularly in hospitals, exacerbating the need for training. Some believed the lack of systematic training in DMC assessments may have contributed to their resistance to DMC. In contrast, primary care participants reported minimal training in health laws, including DMC assessments. They called for closer collaboration with specialist care, preferring concise, case-based training formats. In 2022–23, several participants stressed the importance of integrating the consequences of treatment decisions into systematic DMC assessment training. Moreover, they recommended incorporating this dynamic threshold for DMC (a “sliding scale”) and emphasising sound clinical judgment in the training to improve assessment quality.

## Discussion

### Summary of main findings

Following the introduction of a capacity-based mental health law in Norway in 2017, challenges in DMC assessments emerged, with developments experienced by participants between 2018 and 2022–23. Despite difficulties in its application, the four abilities model was widely used for DMC assessments in specialist care. Over time, a gradual clinical adaptation and decreased importance of DMC assessments were noted. Variations in assessment quality were described across the healthcare system, particularly in primary care. Challenges in clinical practice included applying the four abilities model, lack of continuity of care, information, and cooperation. Certain patient groups, including those with manic symptoms, substance misuse, and severe eating disorders, were regarded as particularly challenging to assess. DMC assessments seem to be frequently conducted with inadequate training and guidance, with limited availability and use of validated tools.

### Challenging to assess DMC and the need for quality assurance

Our findings highlight challenges in applying the four abilities model, which appears widely used for DMC assessments in specialist care. Participants’ experiences with this model, particularly the ability to appreciate, suggest misconceptions about DMC assessments, including that DMC is merely a measure of agreement between clinicians and patients. However, DMC assessments aim to determine whether patients can provide plausible explanations for their treatment choices. Moreover, the lack of assessment structure, including clinicians providing adequate information to the patient before applying the four abilities model, may reduce the quality of DMC assessments – or their reliability and validity [33]. Given its widespread clinical use in Norway, improving the application of the four abilities model seems key to ensuring the assessment quality [33, 34] while incorporating international research for a more systematic assessment approach [10].

Our findings indicate both inadequate quality assurance of DMC assessments and limited awareness of its significance among participants. While some variations in DMC assessments may be expected during their initial implementation, these variations underscore the need for policymakers to prioritise improvements in assessment quality [35]. Variations in the quality of DMC assessments may lead to incorrect involuntary care decisions. Moreover, the lack of professional ownership and limited DMC assessment training may have contributed to inappropriate referrals and inconsistent legal application [35].

Key findings reveal the need for systematic assessment training across primary and specialist care in Norway, aligning with international experiences. A 2020 systematic review by Scott et al. found that practitioners in England and Wales experienced DMC assessments as complex and called for systematic training and increased knowledge about the legal framework [12]. Similarly, a 2020 qualitative study by Vara et al. found limited training and confidence in assessing DMC among general practitioners in New Zealand, requiring tailored education to improve their clinical and legal knowledge and assessment skills [24]. Thus, addressing assessment challenges seems to require systematic training and tailored education in both the clinical and legal aspects of DMC assessments [35, 36].

Findings suggest that resistance to using structured, validated tools for DMC assessments arises from concerns about additional complexity, time constraints, and disruptions in clinical communication. While systematic training could improve the reliability and validity of DMC assessments, the comprehensiveness of structured validated tools appears to be a key barrier to their implementation in clinical practice. Moreover, clinicians seem to spend more time on involuntary care decisions, partly due to several new procedural requirements introduced in the comprehensive 2017 amendments [15], which may further increase resistance to DMC. Attributing challenges that were prevalent before 2017 to DMC may also reflect clinicians’ limited professional ownership of DMC assessments. The inadequate quality assurance measures may be linked to this limited ownership, as well as inadequate training and experience with quality assurance measures among clinicians. Additionally, this may have been accentuated by the insufficient emphasis from Norwegian health authorities on quality assurance of DMC assessments following the 2017 amendments [15].

### Challenges in translating law into clinical practice

Translating DMC as a legal criterion into clinical practice requires addressing both legal ambiguities and assessment challenges. Our findings indicate that when DMC was introduced in the MHCA, it was largely reduced to legal capacity without adequately considering the complex clinical components of DMC assessments. These include, for instance, managing fluctuating patient conditions and addressing doubts about DMC during assessments. A singular focus on legal aspects of DMC risks side-lining complex clinical and ethical considerations, potentially alienating clinicians from their assessment responsibilities. The challenges of translating law into clinical practice are evident in the increasingly comprehensive sections on DMC assessments in the circular to the MHCA, which has been updated in response to Supreme Court cases and other legal developments since 2017. For example, the circular did not adequately address risk sensitivity in assessing the consequences of patients’ choices until 2022 [15, 37–39]. Additionally, the four abilities model is strongly influenced by legal principles, as it is based on analyses of mental health laws and case law [40]. To address some of these assessment challenges, the Norwegian Ministry of Health and Care Services proposed several legislative amendments to the Norwegian Parliament in November 2024 [41]. These amendments included lowering the evidentiary requirement for lacking DMC from “obviously” to “most likely” and incorporating the consequences of patients’ choices into the legal criterion for lacking DMC.

Our findings indicate a shift from initially narrow DMC application to its gradual integration into clinical practice, reflecting clinicians’ ethos of delivering patient care and continuing a “business as usual” approach. This aligns with trends in Norwegian health registry data, which indicate an initial reduction in involuntary care and treatment rates followed by a gradual return to the rising pre-2017 trends [15, 19]. Notably, there was a similar increase in the prevalence of CTOs following the implementation of Queensland’s capacity-based Mental Health Act of 2016, which emphasised the protection of patients’ rights through voluntary care alternatives [21, 22]. Similar to Norway, possible explanations for this unintended consequence in the Australian jurisdiction included a limited understanding of DMC among clinicians and judges, a restrictive, risk-averse culture among clinicians and in society at large, pressure on under-resourced public mental health services, a shortage of voluntary treatment alternatives, and inadequate implementation of the legislative amendments [21, 22]. Influencing clinicians’ decision-making processes remains challenging when they adapt to circumvent legal objectives conflicting with their preferred clinical outcomes [14]. DMC assessment quality forms a cornerstone of sustainable capacity-based systems, requiring time for implementation [15]. Recognising the complexity of DMC seems essential to improve its legitimacy among clinicians and potentially reduce their resistance.

When policymakers aim to influence clinical decision-making through legislation, they must understand the complexities of the healthcare system where clinicians operate. Rather than a well-tended garden trimmed and regulated by politicians and bureaucrats, the health system resembles a jungle [42]. Achieving the long-term objective of reducing involuntary care seems to require addressing social, service, and legal factors [14, 20, 22]. This includes a fundamental shift in how health professionals perceive treatment needs, influencing their clinical and ethical application of the law [43]. Thus, recognising the complex interplay between legal, health service, and societal factors appears crucial for successfully implementing health law reforms and achieving their objectives [22].

### Limitations

One limitation of our study is the difficulty of distinguishing DMC assessments from other aspects of clinical evaluations, leading to uncertainty about when, how, and even if DMC was being assessed. This ambiguity may have challenged participants' ability to respond to questions presupposing a clear distinction between DMC and other evaluation aspects. Despite their own limitations, an observational study design and a qualitative text analysis of DMC assessments in medical records could have provided valuable insights into how DMC assessments are conducted in clinical settings, complementing our findings.

The attrition from 44 participants in 2018 to 21 in 2022–23 is another limitation, partly due to the inclusion criterion of two years of relevant experience to participate in the 2022–23 interviews. The differential attrition among primary care physicians limits perspectives on the legislative amendments across the healthcare system. Moreover, further research is needed to understand patients’ and families’ perspectives on DMC assessments. The limited number of participants in different groups across the four geographic areas also hindered the exploration of geographic variation. Maintaining the same number of participants per group in 2022–23, regardless of previous inclusion, could have prevented this attrition. 

## Conclusions

DMC assessments have posed several challenges in clinical practice following the introduction of a capacity-based mental health law in Norway in 2017. While the four abilities model was widely adopted for DMC assessments in specialist care, variations in DMC assessment quality seem prevalent across the healthcare system. Moreover, the gradual clinical adaptation and decreased importance of DMC assessments seem to have coincided with a lack of ownership of DMC among clinicians. Limited systematic training, a lack of validated tools, and the strict evidentiary requirement of “obviously” lacking DMC may have contributed to more patients being assessed as competent unless they were severely ill, potentially raising thresholds for involuntary care. Similar to other jurisdictions introducing capacity-based mental health laws, the unintended rise in involuntary care rates in Norwegian health registry data since 2017 suggests a shift from voluntary to involuntary care, undermining the amendments’ objectives of strengthening patient autonomy. While it remains to be seen whether the proposed legislative amendments by the Norwegian Ministry of Health and Care Services in November 2024 will be enacted into law, they highlight the ongoing need for effective implementation of Norway’s capacity-based mental health law to balance the aims of strengthening patient autonomy and addressing the need for care. As part of these ongoing implementation efforts, further research is needed to improve DMC assessment quality and better understand the factors influencing these assessments.

## Supplementary Information

Supplementary Material 1.

## Data Availability

The data used and analysed during this study are not publicly available to protect the privacy of participants, in compliance with relevant approvals. Data is provided within the manuscript.

## Appendix

**Table 3:** Themes, subthemes, and illustrative quotes in 2022–23 regarding assessing DMC in clinical practice following the introduction of a capacity-based mental health law in Norway in 2017 (see Table 2 for more illustrative quotes)

Theme	Subtheme	Code	Quote (type of participant, participant number, quotation number)
*1. Evolving practices and variations in DMC assessments*	1 A. The four abilities model as a basis for DMC assessments	Mainly in specialist care	*“We grew very fond of the four abilities model. It was perhaps what we found most useful in acute psychiatry. There have been few occasions when I used the validated tools*,* and they were helpful when I was uncertain about the patient’s DMC.” (Psychiatrist*,* 7*,* Q19)*
	1B. Changes in DMC assessments over time	Gradual clinical adaptation	*“Over time*,* DMC became more established*,* and we gained some experience. Eventually*,* it became less complicated*,* and the current experience is that it has stabilized*,* and it has been incorporated into clinical practice. Now*,* DMC is not really a problem*,* at least compared to how it was initially.” (Primary care participant*,* 23*,* Q20)*
		Decreased importance	*“If you compare DMC assessments conducted from the legislative amendments until now*,* you will observe that they are less comprehensive*,* less thorough*,* and more conclusive. I have also observed a reduction in the level of training in the field compared to the initial stages.” (Psychiatrist*,* 1*,* Q21)*
	1 C. Variations in DMC assessments	Variations in quality	*“There is a significant variation in DMC assessments in primary care. I rarely find that their assessments are well-executed.” (Psychiatrist*,* 2*,* Q22)*
*2. Challenges to DMC assessments*	2 A. Barriers to implementation and quality assessments	Lack of training in applying the four abilities model	*“Each element in the four abilities model is a distinct aspect*,* but they are interconnected. It can be the case that one finds the patient may falter on some of the elements*,* and then it becomes a bit complicated.” (Psychiatrist*,* 5*,* Q23)*
		Lack of ownership	*“Psychiatrists do not have ownership of DMC and tend to view it more as a nuisance than a necessity in our clinical practice.” (Psychiatrist*,* 1*,* Q24)*
		Lack of continuity of care, information, and patient cooperation	*“Instances involving substantial language issues*,* even with an interpreter*,* may pose particular challenges in assessing DMC.” (Psychiatrist*,* 10*,* Q25)*
	2B. Patient groups where assessing DMC is particularly challenging	Manic symptoms	*“I find depressed and the manic patients to be particularly demanding. Some of them have a much better understanding of their own condition than severely psychotic patients. At the same time*,* many of them lack the ability to perceive the consequences of the life they lead*,* such as during manic episodes*,* which I think can be very destructive for them in the long run. It has*,* to some extent*,* been challenging to assess involuntary care in this group before*,* but with the introduction of DMC*,* it became even more difficult.” (Psychiatrist*,* 20*,* Q26)*
		Substance misuse	*“One may lack DMC due to substance use but assessing it can be challenging. This uncertainty persists with patients who engage in more episodic substance use*,* as their cognitive state may vary significantly afterward. Consequently*,* one’s confidence in making such assessments is undermined.” (Psychiatrist*,* 10*,* Q27)*
		Severe eating disorder	*“If one were to take what patients with severe eating disorders say at face value*,* they often have DMC. Therefore*,* one must be much more cautious not to overestimate their ability to make a treatment decision.” (Specialist in clinical psychology*,* 9*,* Q28)*
*3. The need for quality assurance measures in DMC assessments*	3. Training and tools in DMC assessments	Need for training	*“There was clearly a need for training immediately following the legislative amendments. Some individuals may benefit from additional training now. However*,* my impression is that those who are not proficient in DMC assessments may be uninterested in acquiring the necessary skills. Therefore*,* I am uncertain if providing more training would be effective.” (Psychiatrist*,* 3*,* Q29)*
		Need for tools	*“I only use the elements in the four abilities model and try to seamlessly integrate them into the clinical evaluation. I would not want to use a form for these assessments as it becomes too disruptive in the patient interaction.” (Psychiatrist*,* 3*,* Q30)*

## Abbreviations

ACE: Aid to Capacity Evaluation
CTO: Community-Treatment Order
DMC: Decision-making capacity
MacCAT-T: MacArthur Competence Assessment Tool for Treatment
MHCA: Mental Health Care Act
