# Supplementary material for: Assessing decision-making capacity in clinical practice in Norway: a qualitative exploration of stakeholder perspectives
Source: BMC Psychiatry. 2025 Oct 10;25:965. doi: 10.1186/s12888-025-07161-z (PMC12512581; doi:10.1186/s12888-025-07161-z)
Supplement: Supplementary file 1 — Supplementary Material 1. [file 12888_2025_7161_MOESM1_ESM.pdf]

Supplementary Material 1: Interview Guide

Aims

Assess the impact of the legislative amendments, the quality of assessments of decision-making capacity (DMC), and how they are conducted in the context of involuntary observation and care, Community Treatment Orders, and involuntary treatment. The term "quality" refers to whether the DMC assessments are good and accurate (validity).

Questions for all participants

1. *What are your experiences with the impact of introducing DMC as part of the amendments to the Mental Health Care Act in 2017?*

- For example, what consequences (positive and negative) has the DMC criterion had?
- For example, increased autonomy and dialogue, or whether the DMC assessment is perceived as demeaning?
- Reduced involuntary care (if so, what types)?
- Consequences for informal involuntary care, patient deterioration, readmissions, time spent (documentation, dialogue, follow-up), requirements for collaboration, burden on families?

2. *Do you have other experiences of how DMC assessments are conducted after the legislative amendments?*

Questions for clinical and supervisory body participants

3. *How is DMC assessed? How do you evaluate the quality of the assessments? Are there any challenges related to these assessments? Do you find that DMC is assessed similarly?*

4. *What is the need for training and tools to assess DMC?*

5. *How have the legislative amendments influenced your practice in other areas of health law?*

- For example, in cases of doubt regarding DMC, is involuntary observation used?
- Emphasis on the patient's wishes and experiences, altered use of the danger criterion?
- Is DMC assessed when patients collaborate, or families are involved?
